# Computed tomography scan based prediction of the vulnerable carotid plaque

**DOI:** 10.1186/s12880-017-0233-5

**Published:** 2017-12-13

**Authors:** Hadi Mahmoud Haider Diab, Lars Melholt Rasmussen, Stevo Duvnjak, Axel Diederichsen, Pia Søndergaard Jensen, Jes Sanddal Lindholt

**Affiliations:** 1 Department of Cardiothoracic and Vascular Surgery, Odense University Hospital, Sdr. Boulevard 29, 5000 Odense C, Denmark; 2 Department of Clinical Biochemistry and Pharmacology, Odense University Hospital, Sdr. Boulevard 29, 5000 Odense C, Denmark; 3 Department of Radiology, Odense University Hospital, Sdr. Boulevard 29, 5000 Odense C, Denmark; 4 Department of Cardiology, Odense University Hospital, Sdr. Boulevard 29, 5000 Odense C, Denmark

**Keywords:** Carotid stenosis, Computed tomographic angiography, Histological components, Semi-automated CTA software, Calcifications score

## Abstract

**Background:**

Primary to validate a commercial semi-automated computed tomography angiography (CTA) –software for vulnerable plaque detection compared to histology of carotid endarterectomy (CEA) specimens and secondary validating calcifications scores by in vivo CTA with ex vivo non-contrast enhanced computed tomography (NCCT).

**Methods:**

From January 2014 to October 2016 53 patients were included retrospectively, using a cross-sectional design. All patients underwent both CTA and CEA. Sixteen patients had their CEA specimen NCCT scanned. The semi-automated CTA software analyzed carotid stenosis using different HU values defining plaque components. The predictive values of CTA based detection of vulnerable plaques were calculated. Quantification of calcifications on CTA using region of interest (ROI)-function and mathematical equations was done manually, and validated by NCCT of the CEA specimen.

**Results:**

The semi-automated CTA software had a sensitivity, specificity, positive predictive value (PPV) and negative predictive value (NPV) of 89.1% (95% CI, 73.6% - 96.4%), 31.3% (95% CI, 12.1% - 58.5%), 75% (95% CI, 59.3% - 86.2%) and 55.6% (95% CI, 22.6% - 84.6%). Strong correlation between in vivo CTA and ex vivo NCCT in quantification of calcification was observed, but CTA systematically underestimated calcificationsscore (CALS) with increasing calcification.

**Conclusion:**

The CTA-software cannot be used in risk assessment of patients, due to poor specificity and NPV. The correlation between in vivo CTA and ex vivo NCCT was strong, proposing it to be used in both scientifically and clinical settings, but studies with larger sample sizes are needed.

## Background

Cerebral stroke is a well-known cause of mortality and morbidity primarily affecting the lives of the elderly population worldwide [1]. Stenosis of the carotid artery presents one of the major sources to cerebral stroke. Duplex ultrasound and Computed Tomographic Angiography (CTA) is used pre-operatively, to evaluate the degree of carotid stenosis (CS) and combined with cerebral symptomology to decide whether the carotid endarterectomy (CEA) is beneficial or not. According to European Carotid Surgery Trial [2] prophylactic CEA is beneficial when the symptomatic CS is 70% or greater. However, the risk of stroke or mortality within 30 days of prophylactic CEA is 2.5% and 1% respectively [3]. Furthermore, cerebral stroke can be caused by other diseases in coexistence with CS. Due to those risks, a better selection of patients to CEA is warranted.

CTA is a noninvasive and widely used to confirm severity of CS and accessibility by CEA [4]. In addition CTA software’s have been developed to detect vulnerable plaques with high risk of recurrent cerebral attacks. However, the evidence of the usefulness in these methods are conflicting but still commercial available softwares are offered without proven validity [5, 6].

The role of calcification in plaque stability is still uncertain. A histopathological study [7] subdivided calcifications into macrocalcifications and microcalcifications and concluded macrocalcifications functions as a plaque stabilizer while microcalcifications instabilizing the plaque. Conversely, investigators [8–10] using CTA found the volume of plaque calcifications was higher in asymptomatic cases. However, this has not been confirmed in symptomatic cases, which in many centres are the only indication for CEA and whether CTAs can be used for calcification quantification is controversial. In addition, CTA can’t detect microcalcifications which could be an undetected risk factor for plaque instability.

Consequently, this study aimed primary to validate the ability of a semi-automated CTA-software to discriminate between vulnerable and stable plaques compared to the histology of CEA specimens as a golden standard. Secondary, to investigate if microcalcifications and macrocalcifications contribute to plaque stability or instability. Thirdly to examine whether plaque calcification estimated by in vivo CTA is comparable with plaque calcification estimated by ex vivo non-contrast enhanced CT (NCCT).

## Methods

### Patient population

From January 2014 to October 2016 61 patients were enrolled retrospectively using a cross-sectional study design. Inclusion criteria were: (1) patients diagnosed with Amaurosis Fugax, Transit Ischemic Attack (TIA), minor and/or major stroke; (2) preoperatively CTA scan; (3) underwent CEA.

Amaurosis Fugax is defined as a transit loss of vision on one or both eyes and lasts for a few seconds. TIA as neurological symptoms that disappear within 24 h of onset, and cerebral stroke defined as neurological symptoms that remain after 24 h of onset. As a standardized protocol in our institution, all patients who were admitted were examined neurologically. Symptoms and risk factors were reported. From the patients’ medical records we noted the medical history, medication use, side of the symptomatic carotid artery and neurological symptoms (Table 1).

**Table 1:** Baseline characteristics of included patients stratified by presence of intraplaque haemorrhage (IPH) in the removed stenotic carotid plaque

	*IPH-containing (n = 37)*	*Non IPH-containing (n = 16)*	*P- value*
Age, y, mean (SD)	70.19 (± 9,58)	70.13 (± 7.32)	.816
Sex:			
- Male, % (*n*)	67.6 (25)	31.2 (5)	<.0001
- Female, % (*n*)	32.4 (12)	68.8 (11)	<.0001
Medical History:			
- Hypertension, % (*n*)	56.8 (21)	81.3 (13)	.091
- Diabetes mellitus, % (*n*)	10.8 (4)	37.5 (6)	.024
- Aortic valve disease, % (*n*)	2.7 (1)	0 (0)	.511
- Aortic valve operated, % (*n*)	0 (0)	0 (0)	1.00
- Atrial fibrillation, % (*n*)	10.8 (4)	6.3 (1)	.605
- Smoking, % (*n*)	37.8 (14)	56.3 (9)	.219
- Interval (ds)^a^, mean (SD)	17.92 (± 9.57)	22.38 (± 20.15)	.869
Medication use:			
- Statins, % (*n*)	43.2 (16)	62.5 (10)	.202
- Vitamin K antagonists, % (*n*)	8.1 (3)	0 (0)	.245
- Antiplatelet agents, % (*n*)	32.4 (12)	62.5 (10)	.043
- Antihypertensiva, % (*n*)	51.4 (19)	81.3 (13)	.043
Symptomatic carotid artery			
- Left, % (*n*)	37.8 (14)	50.0 (8)	<.0001
- Right, % (*n*)	62.2 (23)	50.0 (8)	<.0001
Neurologic symptomatology:			
- TIA, % (*n*)	59.5 (22)	37.5 (6)	<.0001
- Stroke, % (*n*)	27.8 (10)	50.0 (8)	<.0001
- Amourasis Fugax, % (*n*)	13.5 (5)	12.5 (2)	.571

^a^Interval: Days between 1. event and surgery

### Histological protocol

Immediately after CEA surgery, carotid specimens were placed and kept in ice cold Hanks buffered salt solution containing 10 mM HEPES (both Biological Industries, Beit Haemek LTD, Israel).

One day after surgery, the intact CEAs were sliced into 2 or 5 mm thick slices using a custom-made slicing device. The slices were numbered chronologically starting caudally. Every third slice, beginning at slice number 2, was formalin fixed in 4% Paraformaldehyde (4% PFA) for 3-5 days and paraffin embedded. In average, 2 slices (minimum 1 slice and maximum 5 slices per CEA specimen) from a CEA specimen were fixed and analyzed histologically.

Sections 3 μm thick, were used for general histology staining while sections 2 μm thick, were used for immunohistochemically staining.

Stainings were done systematically in the following way: (1) Masson’s trichrome; (2) Von Kossa.

This protocol was performed at Department of Clinical Pathology (Odense University Hospital).

Stained sections were scanned using NPD.view 2 version 2.4 (Hamamatsu Photonics, 812 Joko-cho, Higashi-ku, Hamamatsu city, Japan).

#### Acute intraplaque hemorrhage

We define acute intraplaque haemorrage (IPH) with the presences of erythrocytes in any slice of the CEA specimen. Sections were stained with Masson’s trichrome for identification of erythrocytes [11] (Fig. 2a-c). A nominal scale, consisting of score 0 or 1 was used to indicate IPH or not. Score 0 indicates no presences of erythrocytes while score 1 indicates presences of erythrocytes. IPH-containing plaques were considered vulnerable while non IPH-containing plaques were considered stable. The vulnerable and stable plaques were used as the golden standard when measuring the accuracy of the semi-automated CTA-software.

#### Micro- and macrocalcification

**Fig. 1:**
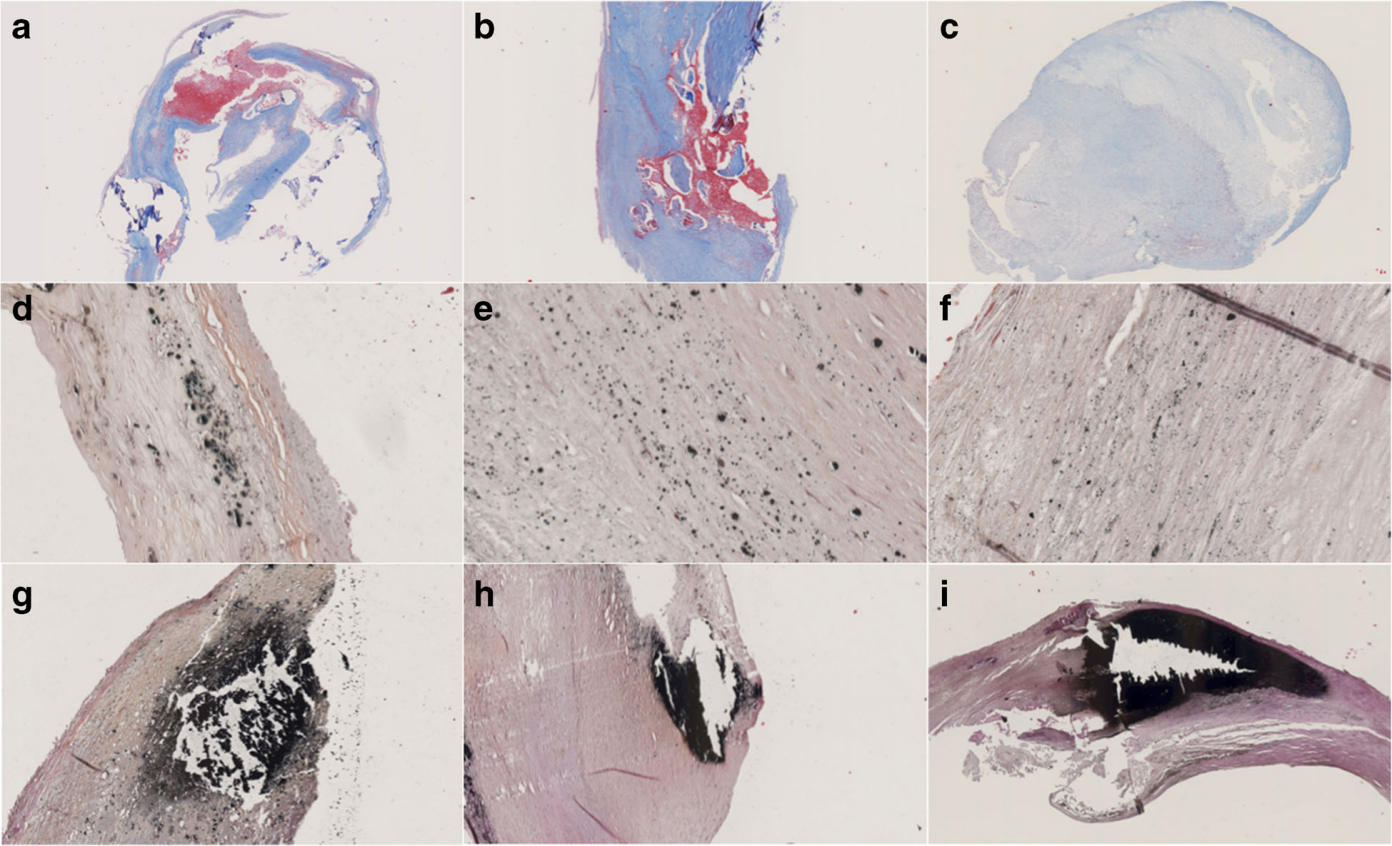
Microscopic apperances (**a-b**) Plaque with IPH (Masson’s trichrome, X1.25 and X10). **c** Plaque without IPH (Masson’s trichrome, X1.25). **d** Microcalcifications clusters (Von Kossa, X20). **e** Single microcalcifications (Von Kossa, X40). **f** Spotty-like microcalcifications (Von Kossa, X40). **g-i** Macrocalcifications (Von Kossa, X10 and X5) (**j-l**)

Microcalcifications are defined as small clusters, single or spotty-like, clusters or single calcifications (Fig. 1d-f), while macrocalcifications are defined as big sheet-like calcifications (Fig. 1g-i) as described [7] but modified. Sections were stained with Von Kossa [7]. We distinguished between micro- and macrocalcifications based on size of the calcifications. Usually, the size of macrocalcifications is bigger compared to the size of microcalcifications. The severity of micro- and macrocalcifications were graded using an ordinal scale which consists of scores from 0 to 3. The scores are based on the percentage of micro- or macrocalcifications covering the plaque area. Score 0 represent plaques not containing micro- or macrocalcifications (0% of plaque area), score 1 represents a mild content of micro- or macrocalcifications (1%-33% of plaque area), score 2 represents a moderate content of micro- or macrocalcifications (34%-66% of plaque area), and score 3 represents a severe content of micro- or macrocalcifications (67%-100%). All sections from a CEA specimens were graded and the maximum score value noted.

### In vivo CTA protocol

CTA was performed on several GE CT scanners including Discovery HD-750 64 slice, Lightspeed VCT XT 64 slice and VCT 64 slice (GE Medical Systems, Little Chalfont, Buckinghamshire, United Kingdom) and on Siemens CT scanners including SOMATOM force and SOMATOM Definition flash (Siemens Healthcare GmbH, Munich, Germany).

Nonionic contrast (80-100 ml of Optiray 350 Covidien, Mallinkrodt Inc., Hazelwood, MO 63042 USA) was injected into the antecubital vein with a rate of 4 ml/s using a power injector. The image acquisition was initiated, when the track bolus reached the aortic arch, controlled by Smartprep (GE Medical Systems, Little Chalfont, Buckinghamshire, United Kingdom).

A caudocranial scanning direction was used covering from aortic arch to approximately 2 cm under the vertex.

### In vivo CTA image analysis

CT images were transferred to AW Server version 2.0 (GE Medical Systems, Little Chalfont, Buckinghamshire, United Kingdom) to analyze the images using Autobone Xpress Neck function.

#### Semi-automated software for plaque analysis

**Fig. 2:**
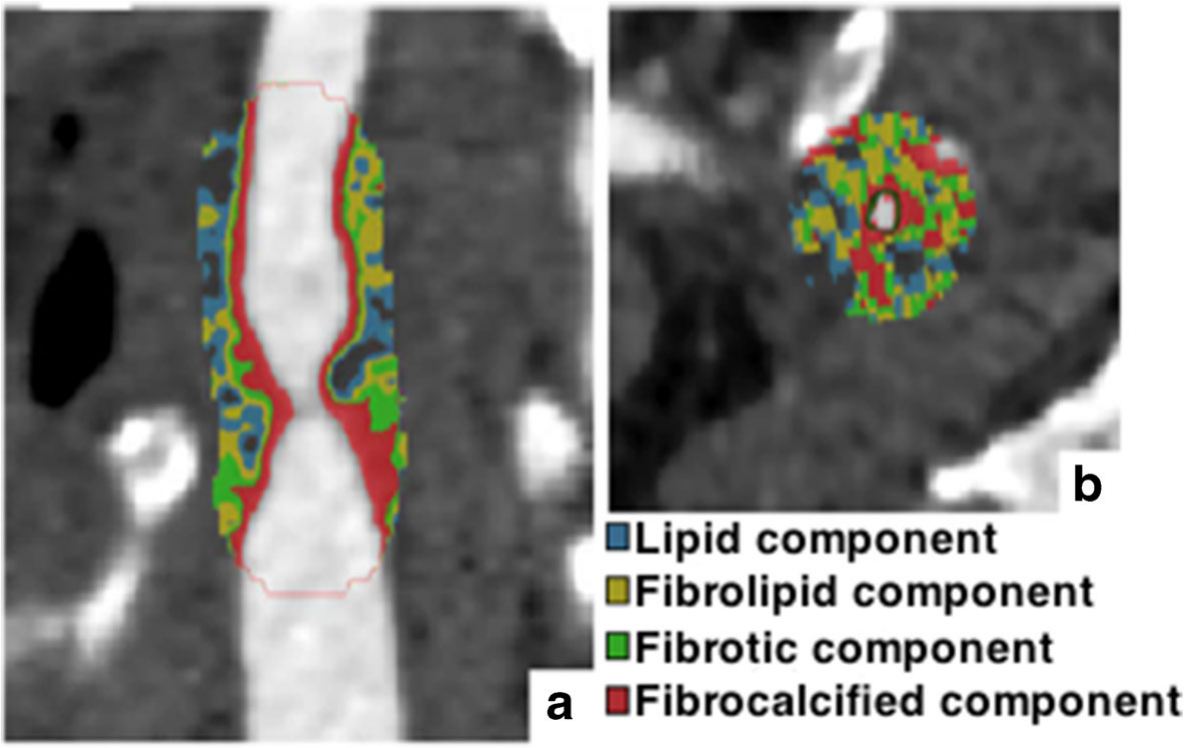
Appearances of a vulnerable plaque using semi-automated software on CTA. a Sagittal section of carotid artery showing the severity of CS. b Transverse section showing the reduced lumen of the vessel. The colours illustrate the distribution of various histological components of the CS based on different HU values, which is automatically calculated by the software

The semi-automated CTA software by GE Medical Systems works by using two functions, for plaque analysis. First, QuickAVA-function was used which makes it possible to choose and analyze one vessel. Secondly, coloridentification-function was used working by setting two endpoints analyzing the preferred area of the carotid plaque using defined Hounsfield units (HU) (Fig. 2). The HU values used was, lipid component 20-39HU, fibrolipid component 40-59HU, fibrotic component 60-79HU, fibrocalcificed component 80-250HU and calcification >250HU (not shown) [5, 12].

The lipid and fibrolipid components are defined as features of vulnerable plaque. A fibrotic component is defined as a feature of stable plaques, but if lipid or fibrolipid components occur then, it becomes a feature of vulnerable plaques.

Fibrocalcified and calcification are defined as stabilizing features. The dominated plaque component decides whether it’s a vulnerable of stable plaque. However, if the carotid vessel is ulcerated [13] the plaque is counted vulnerable.

#### Calculation of the calcifications score (CALS)

Calculation of the calcifications score (CALS) was done manually using a protocol as followed: Area of the calcified lesion, with CT-number > 130 HU were marked using free-hand region of interest (ROI) and measured. The CT-number for each calcific lesion was determined as followed: 1 if peak HU was 130-200HU; 2 if 200-300HU; 3 if 300-400HU; 4 if >400HU. Then, CALS is calculated for each calcific lesion by multiplying the area of the calcific lesion with the CT-number. Calcified lesions with an area under 1mm^2^ were ignored. All CALS were added together, and the CALS for the whole plaque was found. The above measurements were performed using axial slice with a thickness of 0.625 mm, and to convert this to a thickness of 3 mm we finally adjusted the CALS for the whole plaque by multiplying 0,625/3 [14].

### Ex vivo NCCT protocol

After CEA, an ex vivo NCCT scanning was performed of the carotid specimens the same day as surgery. A Siemens SOMATOM Definition Flash (Siemens Healthcare GmbH, Munich, Germany) was used, and the protocol was as followed: Gantry rotation time 0.28 s, 0.625 mm collimation, acquisition 128 × 0.6 mm, 120 kV tube voltage, 90 mAs tube current.

### Ex vivo NCCT image analysis

The CT images were transferred to a workstation, and a dedicated software program Syngo.via (Siemens Healthcare GmbH, Munich, Germany), were used to analyze the scans. Each calcific lesion, with HU >130 was marked and the CALS was calculated. Finally all scores were multiplied and the total CALS for the specimen was automatically calculated.

### Statistical analysis

Sensitivity, specificity, positive predictive value (PPV) and negative predictive value (NPV) were calculated for the semi-automated CTA-software compared with the histological finding of IPH or not. Mann-Whitney U test were used when comparing histology between the two groups with and without IPH. Spearman correlation and Bland-Altman plot were used to validate the association for CALS between NCCT and CTA. To test the reproducibility of the histological analysis protocol and semi-automated CTA-software, Cohen’s kappa coefficient was used. Results were considered significant at *P* < .05. SPSS software version 24 (IBM Corporation, New York, USA) was used for statistics.

## Results

### Patient population

We included 53 patients but 8 patients (15%) were excluded for not fulfilling the inclusion criteria. One had MR-angiography instead of CTA, two had no available data, one had CEA surgery due to hypoperfusion syndrome instead of cerebral stroke and four had no CTA scan due to low eGFR. Sixteen (30%) patients had their CEA specimens NCCT scanned postoperatively.

The patient population was stratified into two groups, an IPH-containing group consisting of 37 patients (69.81%) with a mean (SD) age of 70.19 (±9.58) and a non IPH-containing group consisting of 16 patients (30.19%) with a mean (SD) age of 70.13 (±7.32). The baseline characteristics are summarized in Table 1.

### Accuracy of the semi-automated CTA-software

The efficiency of the semi-automated CTA-software was compared with carotid plaques, with and without IPH as the golden standard. We observed a sensitivity of 89.1% (95% CI, 73.6% - 96.4%), a specificity of 31.3% (95% CI, 12.1% - 58.5%), a PPV of 75% (95% CI, 59.3% - 86.2%) and the NPV was 55.6% (95% CI, 22.6% - 84.6%).

### Comparison of histological components

**Fig. 3:**
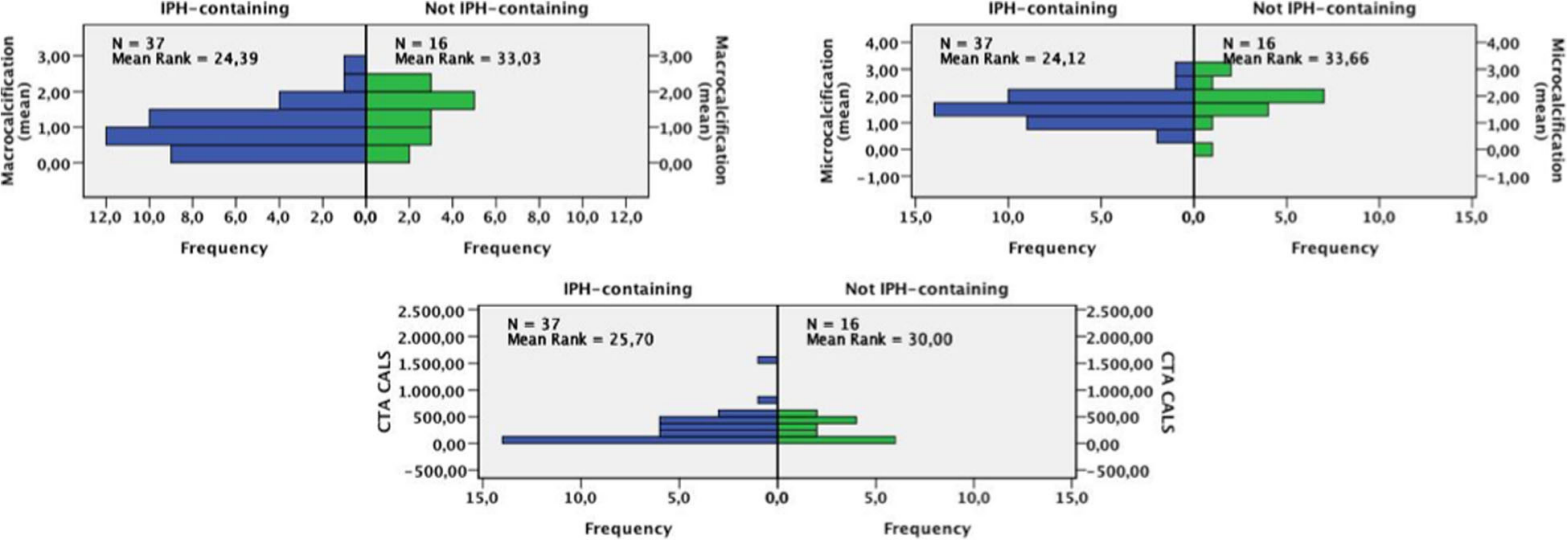
The severity of macrocalcifications, microcalcifications and CTA CALS was compared between the IPH containing group and the non IPH-containing group. Significant difference was only observed for microcalcifications (*P* = 0.034)

Figure 3 shows the comparison of histological calcifications between IPH-containing plaques and non IPH-containing plaques. The non IPH-containing plaques containing surprisingly more microcalcifications compared to IPH-containing plaques (mean rank = 24.11 vs. 33.69, *P* = 0.034), meanwhile no significant was observed for macrocalcifications (mean rank = 24.39 vs. 33.03, *P* = 0.057) and CALS of CTA (mean rank = 25.70 vs. 30.00, *P* = 0.352).

### Association between CALS for CTA and NCCT

**Fig. 4:**
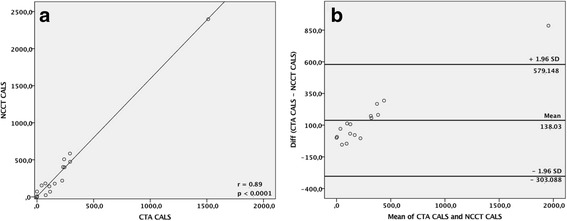
**a** Graph indicating strong positive linear correlation in measuring CALS between CTA and NCCT. (**b**) Bland-Altman plot. X-axis shows the mean calcification score of the two methods. Y-axis shows the difference of the calcification score measured by the two methods. The red line indicates mean difference, black lines indicate the 95% confidence levels of the mean. Circle dots indicates mean values for CALS between CTA and NCCT. If no association between the difference of the measurements existed, the dots should be placed on the redline, but here a clear tendency of increased disagreement with increasing calcification is observed

In 16 patients CTA and NCCT images of carotid plaques were available for comparison. Figure 4a shows a strong correlation of CALS between CTA and NCCT with a Spearman’s Rho correlation coefficient of 0.89 (*P* < 0.001). The Bland-Altman plot (Fig. 4b) indicates when the CALSs were small, i.e. less calcified lesions, the difference in calcification measurement between CTA and NCCT were minor. But when the value of CALSs increases, i.e. severe calcified lesions CTA underestimated the CALSs thereby making the difference between the CALSs larger.

### Reproducibility of the histology and semi-automated CTA-software method

Two observers graded 15 histology sections and their results were compared. The interobserver variation for IPH was 0.60. For macrocalcifications and microcalcifications it was 0.88 and 0.69.

The intraobserver variation of the semi-automated CTA-software was 0.41.

## Discussion

The commercial available semi-automated CTA-software showed unacceptable predictive values regarding vulnerable plaque defined as IPH containing plaques and poor intraobserver agreement. Secondly, microcalcifications were associated with plaque stabilization. However, microcalcifications is not visible at any kind of clinical CT, and macrocalcifications had no association with plaque stability, strong correlation of CALSs between CTA and NCCT was observed.

### The semi-automated CTA-software

Many semi-automated CTA-software’s have been developed for assessing histological components of the carotid plaque as a complement to the degree of the CS, some of these are commercialized. Such products ought to be validated before given free for clinical use, at the consequence of a poor product is potentially invalidating inability or death. Consequently, we validated such a commercialized semi-automated CTA-software which showed specificity and NPV which hasn’t been better than throwing a coin. Furthermore, an intraobserver variation of 0.41 was observed. This is at the border of being classified as poor based of Fleiss who characterize kappas over 0.75 as excellent, 0.40 to 0.75 as fair to good, below 0.40 as poor [15]. The CTA-software uses a method that may depend on the investigators experience in analyzing and evaluating such CTA images. If the investigator is unexperienced this could affect the interpretation of the plaque analysis which could explain the poor specificity, NPV and variability achieved in this study. However, in daily clinical life, it seems most likely that such time consuming tasks is provided by less experienced observers.

Hetterich et al. [16] reported a good sensitivity (> 80%) and excellent specificity (< 90%) for detection of histological components. Furthermore, the interobserver reproducibility was excellent with excellent intraclass correlation coefficient (0.98 or higher). These results are in disagreement with ours, due to the fact that Hetterich et al. uses more experienced observers (5 and 10 years of experience) which could improve the results and a method that could be more effective in assessing CS.

Due to the influence of observer experience on the results, it shows the need of CTA-software’s that uses more standardized methods, which are not dependent of the investigators experience thereby making the result more reliable.

### The carotid plaque risk factors and plaque stabilizers

No significant associations between vulnerable and stable plaques in the severity of macrocalcifications and CALS were observed. Meanwhile significant differences were found in microcalcifications. The lack of association between macrocalcifications and our two groups is important to notice. Our results suggest macrocalcifications isn’t either a risk factor for plaque rupture or a protective feature. This is in contrast with the findings by Hunt et al. [17], who found that stroke and TIA occurred less frequently in patients with large sheetlike calcifications which suggests macrocalcifications is a stabilizing feature and affords a protective effect. This disagreement could be explained by our study population which consists of purely symptomatic patients and that asymptomatic patients carry a larger calcification burden in general.

Surprisingly, a significant difference was observed for microcalcifications which were associated with stable plaques, suggesting microcalcifications to be a stabilizing feature. A previous study by Virmani et al. [18] found in coronary atherosclerotic plaques, that microcalcifications located in the fibrotic cap increases the risk of plaque rupture significantly compared to microcalcifications located in the lipid necrotic core. In the present study, we did not distinguish between specific areas of the plaque when identifying microcalcifications. Instead microcalcification was evaluated in the carotid plaque in general. The concept of subdividing calcifications into microcalcifications and macrocalcifications is relatively new. How microcalcifications and macrocalcifications develop, and which function they have in carotid atherosclerotic plaques still remains uncertain and need further investigation.

### CALS between CTA and NCCT

To our knowledge this is the first study comparing in vivo CTA and ex vivo NCCT in estimation of the severity of calcifications of carotids. CTA is generally accepted to be unvalid to quantify coronary artery calcifications, where the technique was developed, but the carotid arteries are larger and not influenced by motion artefact, and this may make a difference. We found a strong correlation between ex vivo NCCT and in vivo CTA CALS in the carotid arteries [19, 20], but when the CALSs were very high CTA tend to underestimate the CALSs compared to the NCCT estimates. The reason for this underestimation may be the contrast itself. When visualizing the carotid vessel with contrast, the contrast tends to cause blooming artifacts of the vessel wall, and this may cause difficulties in distinguishing the contrast from the calcifications, thus making it difficult to define the borders of the calcifications. This problem may be exaggerated if the lesions are severely calcified, since these very calcified lesions have HU values resembling the HU values of the contrast. However, it suggests at least it can be used with non-parametric tools, and that transformation may be possible for parametric use. The latter will especially be useful scientifically and perhaps clinically, but measurements form only 16 patients were obtained. This is a major limitation. A much larger sample size is needed to confirm the correlation and provide and equation for transformation of quantities between CTA and NCCT.

### Strength and limitations in our study

We consecutively included symptomatic patients with CS, based on relevant neurologic symptoms, verified CS with CTA and undergoing CEA. However, 15% of those undergoing CEA weren’t included, leaving a minimal risk of selection bias – especially regarding patients with nephropathy. In addition, our study population consists only of symptomatic patients, making it impossible to generalize our results to asymptomatic patients.

Regarding information bias, such bias is possible regarding our histological analysis. IPH was used as a pragmatic definition of a vulnerable plaque due to its strong association to such [11], and the larger observer variation regarding the standard histological definition of vulnerable plaque [21]. This may have classified some vulnerable plaque as stable, but probably not many taken the high proportion of vulnerable plaques by the IPH definition into consideration. However in that case, the bias is towards the null hypothesis, and this may have underestimated the validity of the CT based software but hardly caused the unacceptable values. Further to minimize risks of information bias regarding the secondary endpoints we standardized our method using ordinal scales for histological calcifications (microcalcifications and macrocalcifications) and nominal scale for the presence of IPH or not. Furthermore, to ensure the results of the histological components isn’t by randomness, we tested the interobserver variations, and showed a variation from fair to excellent. Meanwhile, the results of the CTA-software could seriously be influenced by information bias. This was also confirmed by the relatively poor intraobserver variation. It must be considered as a limitation, that we didn’t test the interobserver variation between unexperienced and experienced observers as this could provide further insight in whether the validity of the software would be acceptable in the hands of very experienced observers. Finally, our study population consists of 53 patients, which is a relatively small sample size with limited statistical power increasing risk of type 2 errors. However, even taken this into consideration by exploring the 95% CI for the validity parameters of the software tool, it still suggests unacceptable predictability. To achieve high statistical power and avoid type 2 errors, a larger sample size is needed and warranted.

## Conclusion

Extreme care should be taken when using even commercial available CT technology to assess carotid plaque carotid morphology, and calcifications on CT scans seem not to be associated with stable plaque. However, contrast-enhanced CT scans may be valid for calcification quantification but needs further investigation.

## Abbrevations

CALS: Calcificationsscore
CEA: Carotid endarterectomy
CS: Carotid stenosis
CTA: Computed tomography angiography
HU: Houndsfield units
IPH: Intraplaque haemorrage
NCCT: Non-contrast enhanced computed tomography
NPV: Negative predictive value
PFA: Paraformaldehyde
PPV: Predictive positive value
ROI: Region of interest
SD: Standard deviation
TIA: Transit ischemic attack
